# Supplementary material for: The vault associates with membranes in situ
Source: Nat Commun. 2026 Apr 21;17:3659. doi: 10.1038/s41467-026-71837-7 (PMC13100048; doi:10.1038/s41467-026-71837-7)

Supplementary Material

Supplementary Table 1: STA map information.

**Tomograms used for STA/TM**	318		
**Map type**	Cytosolic vault with C39 symmetry	Membrane-associated vault with C39 symmetry	Cytosolic ribosomes encapsulated by vault
**Final # of particles**	985	14	80
**Resolution (Å) (FSC 0.143)**	29	NA	NA

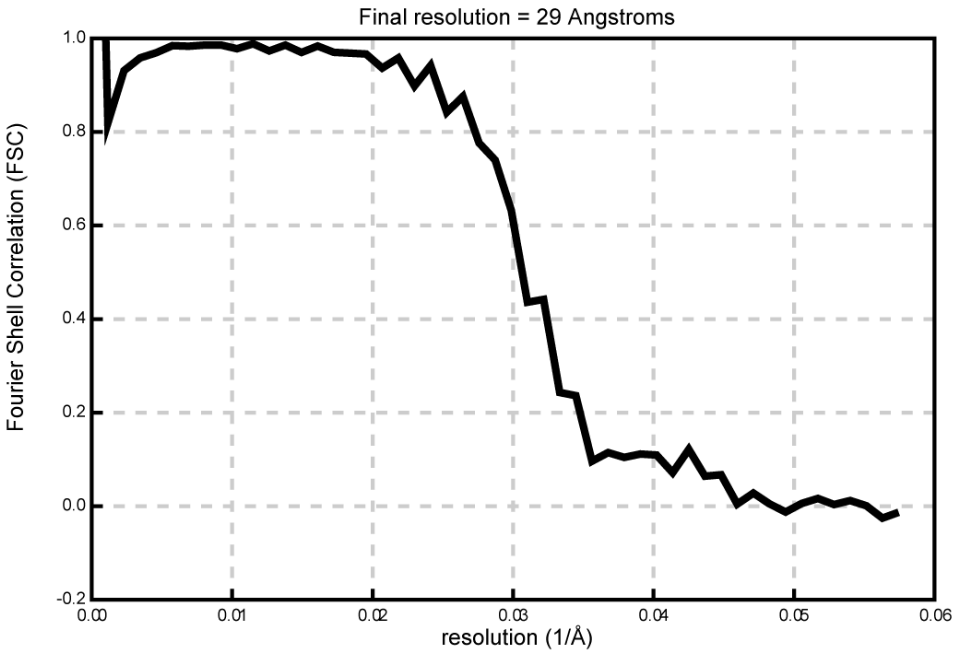

Supplementary Figure 1: FSC curve of cytosolic vault STA as determined by Relion 3.1 postprocessing.

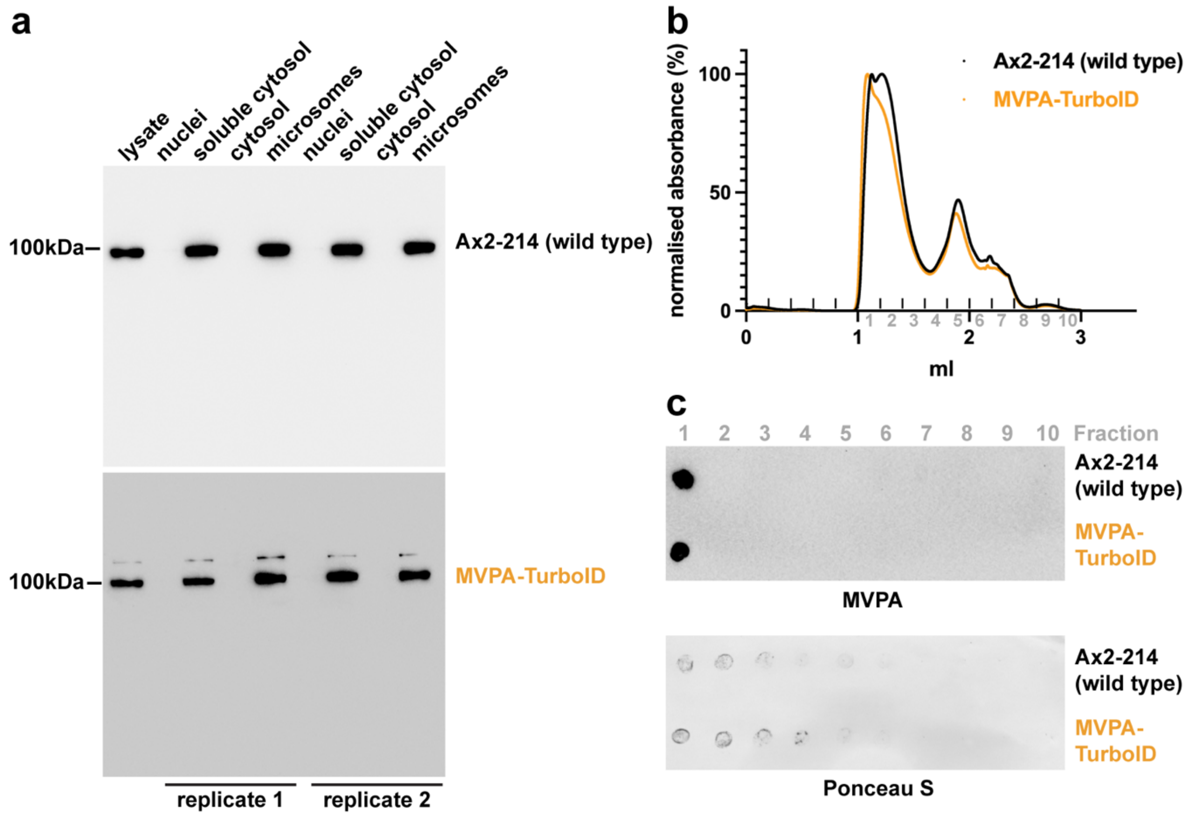

Supplementary Figure 2: Validation of TurboID-tagged vault complex assembly. a: MVPA-TurboID is expressed and incorporated into vault complexes. Both endogenous and modified vaults sediment similarly in subcellular fractionation experiments as assessed by vault (MVPA) Western Blots. Top panel: wild type cell lysate fractionation, endogenous MVPA is detected (94 kDa). Bottom panel: MVPA-TurboID expressing cell lysate fractionation, both MVPA-TurboID (130 kDa) and endogenous MVPA (94 kDa) are detected. N=2 technical replicates. B: Normalized size exclusion chromatography absorbance profiles (Superose 6) of soluble cytosol fractions. Intact vaults migrate with the exclusion volume (1 mL). C: Vault (MVPA) dot blot of the fractions obtained in B. Intact vaults migrate with the exclusion volume (fraction 1) while monomers (1.8-1.9 mL, fraction 5) would elute later.

Supplementary Methods

Validation of vault complex assembly by subcellular fractionation

D. discoideum cells were grown axenically in HL5 medium without glucose (Formedium, supplied with 13.5 g/L glucose, 50 µg/mL ampicillin and 50 µg/mL Hygromycin B for selection) at 21°C/150 rpm as described previously[27]. Cells were pelleted at 400xg for 5 minutes, washed once with 1x KK2 buffer, flash frozen and stored at -80°C. Cell pellets were thawed on ice, resuspended in 1x fractionation buffer (90 mM MES, 10 mM sodium phosphate, 1.5 mM MgCl2, 1 mM EGTA, protease inhibitor (Roche cOmplete, EDTA-free), 1 mM PMSF) supplied with 10% sucrose and 0.05% IGEPAL CA-630 and incubated for 10 minutes on ice. After dounce homogenisation with 20 strokes using a tight pestle, the lysate is incubated for 15 minutes on ice before fractionation.

Fractionation is carried out by differential centrifugation of the lysate first at 10.000xg for 1 hour at 4°C (supernatant: cytosol, pellet: nuclei, intact cells, mitochondria) in a standard tabletop centrifuge. The supernatant is centrifuged at 100.000xg in an ultracentrifuge for 90 minute at 4°C using a TLA55 rotor (supernatant: soluble cytosol, pellet: microsomes and vaults).

0.1% of every fraction is subjected to vault Western Blot (4-12% Bis-Tris on Nitrocellulose, detection using custom rabbit polyclonal MVPA antibody (epitope in shoulder region, antibody generated by Davids Biotechnologie, Germany), secondary antibody: Jackson ImmunoResearch Peroxidase AffiniPure® Goat Anti-Rabbit IgG (H+L) 111-035-003). Both antibodies are used at a dilution of 1:10000. Analyses and fractionations were done in technical duplicates.

Uncropped Western Blots

#	Samples applied	Separation	Membrane	Primary Antibody	Secondary Antibody	Marker
1	Subcell. fractionation, wild type	NuPAGE mini 1 mm, 4-12% Bis-Tris in MOPS SDS	Nitro-cellulose	Rabbit polyclonal *D. discoideum* MVPA (custom)	Peroxidase AffiniPure® Goat Anti-Rabbit IgG (H+L) (Jackson ImmunoResearch)	Precision Plus Protein Dual Color Standard
2	Subcell. fractionation, MVPA-TurboID	NuPAGE mini 1 mm, 4-12% Bis-Tris in MOPS SDS	Nitro-cellulose	Rabbit polyclonal *D. discoideum* MVPA (custom)	Peroxidase AffiniPure® Goat Anti-Rabbit IgG (H+L) (Jackson ImmunoResearch)	Precision Plus Protein Dual Color Standard
3	SEC fractions wild type and MVPA-TurboID	N/A, Dot Blot	Nitro-cellulose	Rabbit polyclonal *D. discoideum* MVPA (custom)	Peroxidase AffiniPure® Goat Anti-Rabbit IgG (H+L) (Jackson ImmunoResearch)	N/A

Western Blot #1

Colorimetric acquisition

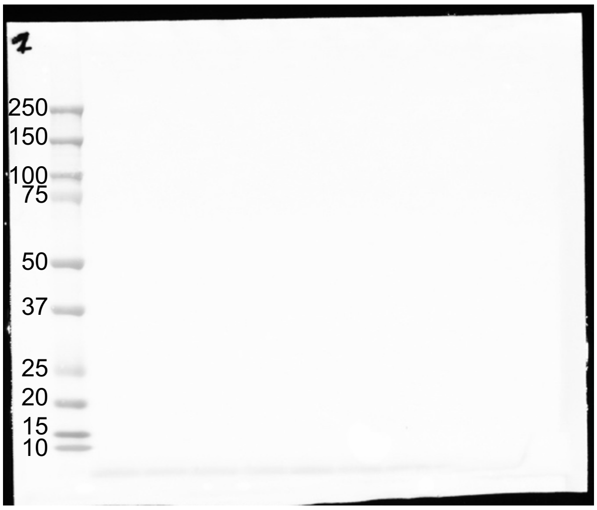

Chemiluminescence detection

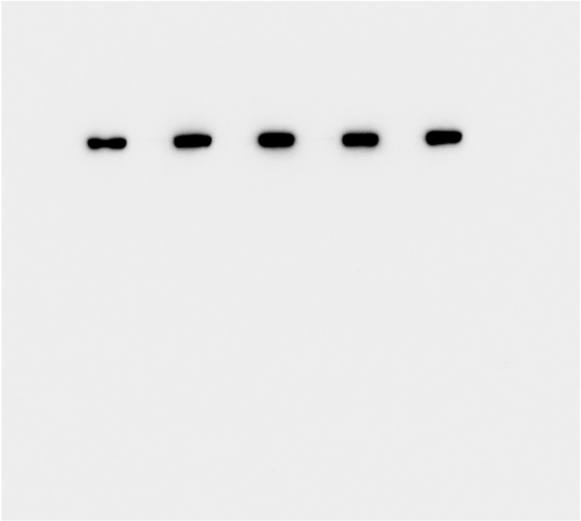

Western Blot #2

Colorimetric acquisition

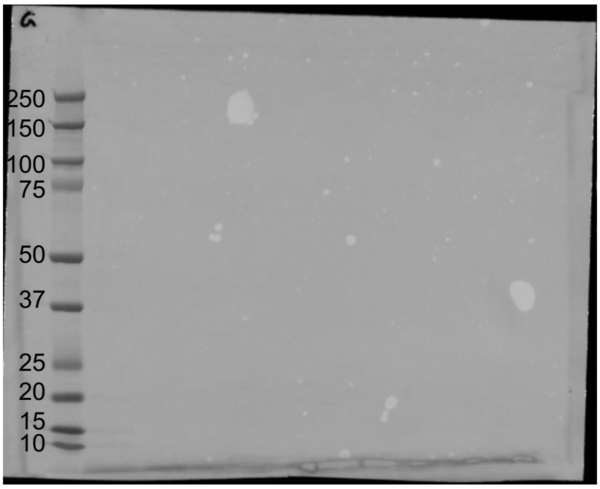

Chemiluminescence detection

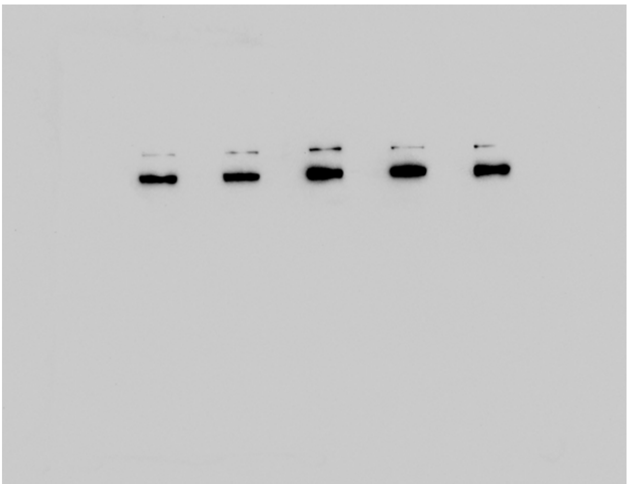

Dot Blot (#3), SEC fractions
Top row: Fractions wildtype 1-10, Bottom row: Fractions MVPA-TurboID 1-10

MVPA detection (chemiluminescence).

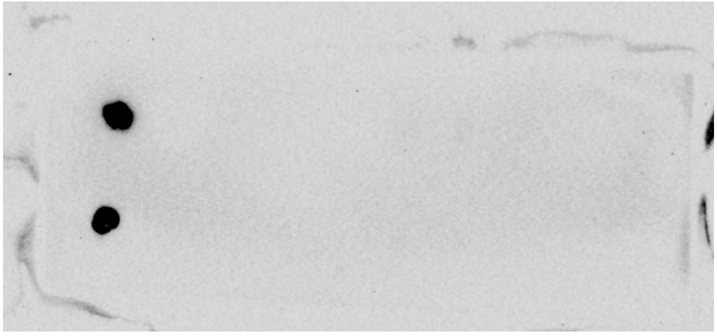

Ponceau S staining (colorimetric detection)

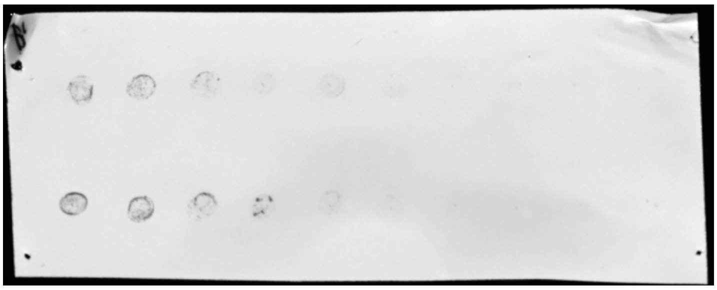

Supplement: Supplementary file 1 — Supplementary Information [file 41467_2026_71837_MOESM1_ESM.pdf]
