# Supplementary material for: The vault associates with membranes in situ
Source: Nat Commun. 2026 Apr 21;17:3659. doi: 10.1038/s41467-026-71837-7 (PMC13100048; doi:10.1038/s41467-026-71837-7)
Supplement: Supplementary file 2 — Reporting Summary [file 41467_2026_71837_MOESM2_ESM.pdf]

Corresponding author(s): Prof. Dr. Martin Beck

Last updated by author(s): Feb 25, 2026

Reporting Summary

Nature Portfolio wishes to improve the reproducibility of the work that we publish. This form provides structure for consistency and transparency in reporting. For further information on Nature Portfolio policies, see our Editorial Policies and the Editorial Policy Checklist.

Statistics

For all statistical analyses, confirm that the following items are present in the figure legend, table legend, main text, or Methods section.

n/a	Confirmed	
☐	☒	The exact sample size (*n*) for each experimental group/condition, given as a discrete number and unit of measurement
☐	☒	A statement on whether measurements were taken from distinct samples or whether the same sample was measured repeatedly
☐	☒	The statistical test(s) used AND whether they are one- or two-sided<br>*Only common tests should be described solely by name; describe more complex techniques in the Methods section.*
☒	☐	A description of all covariates tested
☐	☒	A description of any assumptions or corrections, such as tests of normality and adjustment for multiple comparisons
☐	☒	A full description of the statistical parameters including central tendency (e.g. means) or other basic estimates (e.g. regression coefficient) AND variation (e.g. standard deviation) or associated estimates of uncertainty (e.g. confidence intervals)
☐	☒	For null hypothesis testing, the test statistic (e.g. F, t, r) with confidence intervals, effect sizes, degrees of freedom and P value noted<br>*Give P values as exact values whenever suitable.*
☒	☐	For Bayesian analysis, information on the choice of priors and Markov chain Monte Carlo settings
☒	☐	For hierarchical and complex designs, identification of the appropriate level for tests and full reporting of outcomes
☒	☐	Estimates of effect sizes (e.g. Cohen's *d*, Pearson's *r*), indicating how they were calculated

Our web collection on statistics for biologists contains articles on many of the points above.

Software and code

Policy information about availability of computer code

Data collection	All data was previously published in Hoffmann, Patrick C. et al., acquisition software SerialEM(3.8.1)<br>Molecular Cell, Volume 85, Issue 3, 537 - 554.e11. Data collection is explained in the methods section of the original publication.<br>For proteomics data acquisition, HyStar (6.4) and timsControl (6.1.0.3) was used. UNICORN 7.6 was used to acquire chromatograms. No custom software was used or created.
Data analysis	All software used for data analysis is publicly available in repositories and credited accordingly in the reference section, including version numbers.<br>gctf(1.06), IMOD(version 4.10.9 and 4.11.5), AreTomo (1.33), novaCTF (https://github.com/turonova/novaCTF), novaSTA (https://github.com/turonova/novaSTA), Relion(3.1), STOPGAP(0.7.1), GAPSTOPTM (0.3), ChimeraX (1.8, 1.9., 1.10), cryoCAT, Membrain-seg, Amira Software (Thermo Scientific, ver 2023.2), ArtiaX, napari, FiJi, GraphPad Prism (10.4.), cryoCARE, foldseek (release 10), DIA-NN (2.2.0), MS-DAP (1.2.2.), FragPipe (23,) R (4.3), SWISS-MODEL, ParaView, MSFragger (4.3)

For manuscripts utilizing custom algorithms or software that are central to the research but not yet described in published literature, software must be made available to editors and reviewers. We strongly encourage code deposition in a community repository (e.g. GitHub). See the Nature Portfolio guidelines for submitting code & software for further information.

Data

Policy information about availability of data

All manuscripts must include a data availability statement. This statement should provide the following information, where applicable:

- Accession codes, unique identifiers, or web links for publicly available datasets
- A description of any restrictions on data availability
- For clinical datasets or third party data, please ensure that the statement adheres to our policy

The STA map of the cytosolic D. discoideum vault is deposited in the EMDB with accession code EMD-56516. [https://www.ebi.ac.uk/emdb/EMD-56516]
Additional maps and homology models are available via Zenodo entry 18414818 [https://doi.org/10.5281/zenodo.18414818].
The cryo-ET datasets used in this study are available in the EMPIAR database with accession codes EMPIAR-11845, EMPIAR-11943 and EMPIAR-11944 [https://www.ebi.ac.uk/empiar/EMPIAR-11845/, https://www.ebi.ac.uk/empiar/EMPIAR-11943/, https://www.ebi.ac.uk/empiar/EMPIAR-11944/]
Mass spectrometry proteomics data have been deposited to the ProteomeXchange Consortium in the PRIDE repository104 with the dataset identifier PXD071329 [https://www.ebi.ac.uk/pride/archive/projects/PXD071329]
Source Data are provided with this paper.

Research involving human participants, their data, or biological material

Policy information about studies with human participants or human data. See also policy information about sex, gender (identity/presentation), and sexual orientation and race, ethnicity and racism.

Reporting on sex and gender	NA
Reporting on race, ethnicity, or other socially relevant groupings	NA
Population characteristics	NA
Recruitment	NA
Ethics oversight	NA

Note that full information on the approval of the study protocol must also be provided in the manuscript.

Field-specific reporting

Please select the one below that is the best fit for your research. If you are not sure, read the appropriate sections before making your selection.

☒ Life sciences ☐ Behavioural & social sciences ☐ Ecological, evolutionary & environmental sciences

For a reference copy of the document with all sections, see nature.com/documents/nr-reporting-summary-flat.pdf

Life sciences study design

All studies must disclose on these points even when the disclosure is negative.

Sample size	Sample size for cryo ET data is the size of the published dataset. For proteomics, n=5 was chosen based on similar experiments performed in previous literature and the available time for data acquisition.
Data exclusions	For proteomics data, one replicate (MVPA-TurboID #5) had to be excluded for differential abundance analysis. The sample did not pass quality control criteria with respect to protein abundance distribution. The raw data is provided.
Replication	The TurboID proximity labeling experiment (with n=5 samples) was not replicated due to the elaborate nature of these experiments, the availability of materials and instrument time. The number of replicates is higher than in typical experiments in the field (typical n=3, here n=5).
Randomization	We did not use randomization in our study. The cryo ET dataset was previously acquired and the different datasets were pooled to increase the sample size. Thus, randomisation is not feasible. For mass spectrometry experiments, both TurboID and control cell lines and derived samples had to be prepared in the same way to ensure compatibility of samples and conditions. Mass spectrometry samples were prepared in the same sample preparation run with the same batches of reagents to minimize experimental biases. They were also subjected to the mass spectrometric acquisition on the same occasion to avoid instrument performance-specific variation. Biases would have become apparent during data QC, e.g. during principal component analyses that indicates if other sources of variation other than the analysed proteomic changes were present.
Blinding	For mass spectrometry sample preparation and analysis, blinding is not feasible, as sample traceability during experiments had to be ensured. Since the cryo ET data is pooled, blinding would not have had any effect on the final outcome.

Reporting for specific materials, systems and methods

We require information from authors about some types of materials, experimental systems and methods used in many studies. Here, indicate whether each material, system or method listed is relevant to your study. If you are not sure if a list item applies to your research, read the appropriate section before selecting a response.

Materials & experimental systems

n/a	Involved in the study
☐	☒ Antibodies
☒	☐ Eukaryotic cell lines
☒	☐ Palaeontology and archaeology
☒	☐ Animals and other organisms
☒	☐ Clinical data
☒	☐ Dual use research of concern
☒	☐ Plants

Methods

n/a	Involved in the study
☒	☐ ChIP-seq
☒	☐ Flow cytometry
☒	☐ MRI-based neuroimaging

Antibodies

Antibodies used

Rabbit polyclonal antibodies raised against MVPA-specific peptides and affinity-purified using immobilized MVPA peptides were purchased from Davids Biotechnologie, Germany. Secondary antibody: Peroxidase AffiniPure® Goat Anti-Rabbit IgG (H+L) (Jackson ImmunoResearch), order no. 111-035-003

Validation

The primary antibody was validated in house as follows:
-detection of antigen in lysates separated on Western Blots with correct molecular weight
-affinity purification of affinity-tagged antigen (StrepII-tag) followed by detection with both tag-specific reagent as well as with antibody to be validated
-detection of multiple signals in WBs of lysates of antigen-fusion-protein expressing cells with anticipated molecular weight
-absence of signals at unrelated molecular weight

Plants

Seed stocks

NA

Novel plant genotypes

NA

Authentication

NA
